# What do your eyes reveal about your foreign language? Reading emotional sentences in a native and foreign language

**DOI:** 10.1371/journal.pone.0186027

**Published:** 2017-10-03

**Authors:** Sara Iacozza, Albert Costa, Jon Andoni Duñabeitia

**Affiliations:** 1 Max Planck Institute for Psycholinguistics, Nijmegen, The Netherlands; 2 International Max Planck Research School for Language Sciences, Nijmegen, The Netherlands; 3 Center for Brain and Cognition, Universitat Pompeu Fabra, Barcelona, Spain; 4 Institució Catalana de Recerca i Estudis Avançats (ICREA), Barcelona, Spain; 5 BCBL, Basque Center on Cognition, Brain and Language, Donostia, Spain; Universidad Torcuato Di Tella, ARGENTINA

## Abstract

Foreign languages are often learned in emotionally neutral academic environments which differ greatly from the familiar context where native languages are acquired. This difference in learning contexts has been argued to lead to reduced emotional resonance when confronted with a foreign language. In the current study, we investigated whether the reactivity of the sympathetic nervous system in response to emotionally-charged stimuli is reduced in a foreign language. To this end, pupil sizes were recorded while reading aloud emotional sentences in the native or foreign language. Additionally, subjective ratings of emotional impact were provided after reading each sentence, allowing us to further investigate foreign language effects on explicit emotional understanding. Pupillary responses showed a larger effect of emotion in the native than in the foreign language. However, such a difference was not present for explicit ratings of emotionality. These results reveal that the sympathetic nervous system reacts differently depending on the language context, which in turns suggests a deeper emotional processing when reading in a native compared to a foreign language.

## Introduction

Language is a very powerful tool to communicate emotions. We are often moved when someone tells us a compassionate or a violent story. Similarly, we respond with an automatic emotional reponse when someone insults us or uses taboo words. Beyond a word’s specific meaning, emotional responses may depend on contextual factors. It is likely that because of the emotional social contexts in which these emotional words have been learned, they have priviledged access to the brain circuits associated with emotional processing. In this article, we assess whether emotionally-charged sentences presented in native and foreign languages elicited the same emotional response. This is not the first study exploring this issue. Our novel contribution, however, is the assessment of how the sympathetic nervous system reacts to emotionally related stimuli presented in the native and foreign languages. This reaction will be assessed by measuring pupil dilation.

Pupil dilation has been shown to be sensitive to the effects of valence and arousal, so that pupil sizes are larger in response to valenced and highly arousing stimuli, as compared to neutral stimuli (e.g., [1]). Such reactivity to emotionally-charged content has been proven to be mainly driven by increased activity in the sympathetic nervous system, which in turn mediates the stimulation of the dilator muscle, prompting dilation (see [2] for a exhaustive discussion). This dilation in response to emotional content has been reported to have delayed latencies, so that it starts peaking at around 1 second after the onset of the stimulus (e.g., [1]; [3]). For these reasons, pupil dilation is a promising measure to explore the relationship between language and emotion, and more specifically to test the hypothesis of differential effects between native and foreign language on sentence processing.

According to the *contextual-learning hypothesis* [4], emotional processing is in fact shaped and refined through personal experiences, and the interplay between learning and experience is proposed as a key process in linking verbal information to emotional information (see also [5]). Thus, an individual’s experience with a given language may constrain the vividness of the emotional reaction that the language may elicit. Indeed, it has been suggested that using a foreign language leads to a certain degree of emotional distance, likely driven by the typical learning context of foreign languages (i.e., a mainly formal and neutral context), which differs from that of learning a native language (i.e., a familiar and emotionally-grounded context; see [6]).

Introspective data collected from bilinguals has shown that they report being emotionally affected differently depending on the language they communicated in, with foreign language fostering a certain degree of emotional distance (see [7], for review). Additionally, the presentation of emotional linguistic stimuli in a foreign language, as compared to the native language, has been shown to modulate behavioral, hemodynamic and physiological responses. This has been reflected in reduced attentional shift during single word recognition [8], decreased activation in the emotion network when reading emotion-laden literature [9], and diminished skin conductance responses to taboo words and childhood reprimands [10]. Numerous studies have indeed pointed to the existence of the so-called *Foreign Language Effects*, an umbrella term which initially referred to differences in judgments and decision-making choices due to the language of instruction, but which has nowadays been extended to more general dissimilarities between native and foreign language processing in emotionally-charged contexts (see [11] for a review). Importantly, the self-reported emotional distance is supported by recent experimental evidence showing strong effects of language in higher level cognitive processes usually affected by cognitive biases, like the reliance on heuristics during decision-making (e.g., [12]; [13]), or like the pervasive effects of the self-bias during perceptual matching (e.g., [14]). These findings suggest that, even in contexts in which linguistic information plays a relatively small role (as in [14]), foreign language contexts impact people’s automatic responses to emotionally-charged stimuli.

These data suggest that reading emotionally-charged material in a foreign language could potentially engage the sympathetic nervous system to a weaker extent. However, the aforementioned studies present a fundamental limitation due to the specific type of material used, which relates to individual experience with the linguistic content (as in [10]). By tapping into autobiographical memories, language-independent effects due to memory inequalities could be mistaken as representative of online processing differences between the native and foreign language. Therefore, in order to make stronger conclusions about a reduced involvement of the sympathetic nervous system when reading in a foreign language, carefully designed stimuli need to be used. The current study was designed with this aim.

We asked young adult participants to read neutral and emotionally charged sentences either in their native language (Spanish) or in their foreign language (English). Two main measures were taken. First, as an implicit (automatic) measure of emotional reactivity, we recorded participants’ pupil dilation while reading the sentences. As a more explicit measure, we asked participants to provide subjective ratings of emotional impact after reading each sentence. By comparing these two measures, we explored whether the differential effects that may be found in the foreign language could originate from difficulties in understanding the emotional content of the sentence, potentially due to the lower proficiency in the foreign language than in the native language, and/or by a reduced autonomic reactivity of the sympathetic nervous system that could arise from the emotional distance associated with foreign language processing. We expected to replicate a general effect of emotion with larger pupil dilations in response to the emotionally-charged sentences as compared to neutral ones. Crucially, we expected a significant interaction between emotion and language, such that the magnitude of the effect of emotion would be smaller in the foreign language.

There is, however, another possible prediction that is based on the differences in the cognitive load associated with native and foreign language processing. Given that pupil dilation is also sensitive to cognitive load (e.g., [15]), one could predict a main effect of language, in addition to the effect of emotion. However, these two effects may not interact. As it has been recently shown ([16]), the affective cost of lying and the cognitive load driven by using a foreign language do not necessarily interact when producing deceiving statements in a foreign language. Similarly, the burden of reading in a language different from the native one could be predicted to lead to an overall effect of language, but no differences in how the sympathetic system reacts to emotionally charged sentences between the two linguistic contexts.

## Methods

### Participants

Fifty-four native Spanish speakers with normal or corrected-to-normal vision were tested. All participants had English as a foreign languge and their proficiency was evaluated through several measures (see Table 1). Participants were randomly assigned to the native or foreign language groups. The groups were matched on demographic characteristics, as well as on empathic and intellectual skills (see Table 1), measured by the Empathy Questionnaire (EQ) [17] (see S1 Fig for the Spanish version), and a 6-minute abridged version of the Kaufman Brief Intelligence Test (IQ) [18] respectively. Ethical approval was granted by the Ethics Committee of the Basque Center on Cognition, Brain and Language. All participants provided written informed consent before the start of the experiment.

**Table 1:** Descriptive statistics of the two experimental groups. Standard deviations are provided in parentheses.

	Native Language	Foreign Language
Participants (number)	27	27
Females (number)	18	17
Age (in years)	23.48 (3.25)	23.93 (4.84)
English AoA (in years)	5.96 (2.44)	6.52 (2.77)
Exposure to English (% of the time)	15.56 (8.01)	11.85 (6.23)
Reading in English (% of the time)	17.41 (13.18)	14.82 (13.69)
Self-perception of overall English (1-to-10)	6.85 (1.73)	6.63 (1.71)
English vocabulary (out of 77)	58.67 (7.18)	58.37 (8.36)
English interview (1-to-5)	3.85 (0.72)	3.70 (0.67)
IQ score	25.41 (3.51)	25.11 (3.43)
EQ score	43.89 (8.15)	46.67 (8.17)

Note: No statistically significant differences between groups were observed in any of these individual variables and cognitive measures (all *ps* > .05).

### Materials

#### Critical word selection

The stimuli were selected from a large database of English lemmas [19]according to their values of valence (i.e., pleasantness of the emotion suggested by the word) and arousal (i.e., the degree of arousal evoked by the word), resulting in 244 nouns and adjectives. Two lists were created, one for negative and the other for neutral words. Crucially, negative words differed from neutral items on both valence (*t* = 6.19; *p* < .001) and arousal (*t* = 25.56; *p* < .001) dimensions. In addition, in order to control for potentially confouding effects, frequency values for the target words were collected from N-Watch [20] for the English words, and from B-Pal [21] for the Spanish ones. There were not significant differences in frequency between the two languages (*t* = 0.28, *p* = 0.78) (see Table 2 for details).

**Table 2:** Characteristics of target words.

	Negative words	Neutral words
***English***		
**Valence**	2.42 (0.39)	5.26 (0.28)
**Arousal**	5.35 (0.77)	3.26 (0.46)
**Frequency (Log10-transformed)**	0.89 (0.60)	1.09 (0.68)
***Spanish***		
**Frequency (Log10-transformed)**	0.76 (0.59)	0.97 (0.60)

For English critical words: Means of valence (1-to-9), arousal (1-to-9), and log-transformed frequency values for both Negative and Neutral conditions. For Spanish critical words: Means of log-transformed frequency values. Standard deviations are provided in parentheses.

#### Stimuli

The critical words from the two lists were assembled to form semantically meaningful adjective-noun phrases (e.g., the hostile terrorist) which were further embedded in twenty incomplete sentential frames (e.g., "At noon __ __ will bring __ __ to __ __"). Each sentential frame was used twice, resulting in twenty negative and twenty neutral sentences. In order to maintain the emotional content throughout the whole sentence, the critical phrases were positioned at the beginning, near the middle, and at the end of each line of text. The sentences were then translated to Spanish by two Spanish-English bilinguals. The resulting Spanish sentences were then further normed via an online questionnaire administered to a different group of 25 Spanish native speakers (15 females; mean age 29.76 years old; SD = 5.65) who did not take part in the experiment. The semantic composability of the sentences was tested on a 7-point scale (Q: Does the following sentence make sense to you?; 1: not meaningful; 7: very meaningful). Sentences belonging to the neutral and the negative conditions were equally rated (Neutral: mean = 4.93, SD = 1.63; Negative: mean = 5.00, SD = 1.51; *t* = 1.19, *p* = .43; *β* = .18 (.15)).

A total of 40 sentential items per language across two conditions (negative, neutral) yielded 20 items per participant per condition (see Table 3 for examples).

**Table 3:** Sentence examples per language and per condition.

English Negative	At noon the hostile terrorist will bring his toxic bomb to the schizophrenic cannibal.
**English Neutral**	At noon the civil receptionist will bring his meticulous petition to the municipal doorman.
**Spanish Negative**	Al mediodía el terrorista hostil llevará su bomba tóxica al canibal esquizofrénico.
**Spanish Neutral**	Al mediodía el recepcionista civil llevará su meticulosa petición al portero municipal.

#### Procedure

The experimental session lasted for approximately 45 minutes in total. After the two tasks used to collect individual EQ and IQ scores, participants sat in a dimly lit room. Each participant completed the experiment in the language to which they had been randomly assigned (Spanish or English). They were presented with 40 trials consisting of the presentation of a fixation cross (that also served to perform inter-trial drift corrections compensating for minimal head movements), followed by the presentation of the target sentence for 10s. The fixation cross was placed vertically centered at the left side of the display, coinciding with the precise location of the first letter of the first word of each sentence. This was done this way in order to minimize potential differences between the initial gaze position across participants and trials. Participants were instructed to read the sentences aloud at a natural pace for comprehension. After each sentence, instructions appeared asking participants to rate on a 1-to-7 scale the emotional impact of the sentence (1 = low, neutral impact; 7 = high, negative impact). These instructions were given in the language in which the experimental session was conducted. Emotional impact ratings were collected by pressing the appropriate button on a response box with 7 labelled buttons located in front of them at a comfortable distance.After familiarization, the proper experiment was administered. Each sentence was presented in the two versions (i.e., negative and neutral) for a total of 40 trials. Trial presentation was randomized across participants. Materials were presented using Experiment Builder (SR Research, Ontario, Canada) on a 19-inch CRT screen (1024x768 at 100Hz) which also served to collect the rating responses and to monitor the right pupil of the participantsat a rate of 1000Hz using an SR Research EyeLink 1000 eye-tracker.

#### Data analysis

Two dependent measures were collected: the subjective ratings about the perception of the emotional impact evoked by the sentences and the pupil size averaged across the 10-second trial, computed for each participant in each trial. The reason for having such a long time-window measure was to explore pupil dilations induced by the emotional status of the sentences as a whole (see [22]; [23] for long time-window analyses). As mentioned above, the stimuli were in fact created by embedding several target words which were distributed all along the sentences (i.e., at the beginning, the middle, and the end). This was done in this way to maximize the effect of emotional content on pupil dilation, and to minimize potential effects due to differences among single words within each sentence. Prior to analysis, a data-cleaning process was carried out on the pupil data. Trials in which the pupil size values deviated more than 2.5 standard deviations (SDs) from the mean for each participant in each condition were considered outliers and were subsequently discarded from the analysis (0.97% of the trials). Furthermore, two participants were completely removed from the pupil analysis because their mean pupil size in both conditions deviated more than 2.5 SDs from the population means, resulting in fifty-two final participants. The remaining data, including emotional ratings, were analyzed using sum-to-zero coding in a series of linear-mixed effect models using the *lmer* function of the *lme4* package (version 1.1–12; [24]) in R (version 3.3.1; [25]). P-values of the effects were determined by computing Type III parametric bootstrapped tests as implemented in the *mixed* function of the package *afex* [26], which in turn calls the function *PBmodcomp* of the package *pbkrtest* [27]. We used the function *lsmeans*, from the homonymous package [28], to explore the interaction effects. This function tests linear contrasts among predictors and automatically applies Tukey p-value corrections.

## Results

### Ratings

A linear mixed-effect model was used to analyze whether the subjective ratings were affected by our manipulations. Therefore, ratings of emotional impact were set as the dependent variable, whereas Emotion (Negative|Neutral), Language (Native|Foreign) and their interaction were treated as categorical predictors. Furthermore, to control for the potential effects of sentence composability and target word frequencies, we added two covariates: a) a measure of composability, obtained from the norming, b) and the mean frequency per sentence, calculated by averaging the log10-transformed values of the target words embedded in each sentence. Both measures were centered, as is customary. Regarding the random structure of the model, we included a per-participant and a per-item random adjustment to the fixed intercept, as well as a slope for Emotion by participant.

The model showed a significant overall effect of Emotion (*t* = 20.64, *p* < .001; *β* = 1.83 (0.09)): negatively charged sentences elicited significantly higher emotional ratings (mean = 5.43, SD = 1.40) than neutral sentences (mean = 1.76, SD = 1.10). The main effect of Language was not significant (*t* = 0.42, *p* = .68; *β* = 0.04 (0.10)), nor the interaction between Language and Emotion (*t* = 0.25, *p* = .80; *β* = 0.02 (0.08)), showing that the explicit perception of sentence emotionality was highly similar in the native and foreign language contexts (see Table 4 and Table 5A for the model’s syntax and results).

**Table 4:** Means of the dependent measures per condition.

	Negative sentences	Neutral sentences	Emotion Effect
**All participants**			
**Pupil size (arbitrary unit)**	1409 (345)	1394 (344)	15
**Emotional ratings**	5.43 (1.40)	1.76 (1.17)	3.67
**Native Language**			
**Pupil size (arbitrary unit)**	1414 (380)	1392 (381)	22
**Emotional ratings**	5.37 (1.42)	1.74 (1.10)	3.63
**Foreign Language**			
**Pupil size (arbitrary unit)**	1403 (306)	1397 (304)	6
**Emotional ratings**	5.49 (1.38)	1.77 (1.13)	3.72

Means of the dependent measures in the Negative and Neutral Sentence conditions together with the Emotion Effect (i.e., Negative *minus* Neutral) in each Language group. Standard deviations are provided in parentheses.

**Table 5:** Lmer models' syntax and results.

a. Lmer (Emotional ratings ~ Emotion * Language + Sentence Composability + Averaged LogFrequency + (1+Emotion|participant) +(1|item))				
	Β	SD	t-value	p-value
Intercept	3.59	0.10	36.44	<0.0001
Emotion	1.83	0.09	20.64	<0.0001
Language	0.42	0.10	0.42	0.68
Emotion x Language	0.21	0.08	0.25	0.80
Sentence Composability	0.25	0.82	0.30	0.76
Averaged LogFrequency	-0.07	0.25	-0.29	0.77
b. Lmer (Pupil size ~ Emotion * Language + Sentence Composability + Averaged LogFrequency + (1|participant) + (1|item))				
Intercept	-0.03	0.02	-1.28	0.20
Emotion	0.11	0.02	4.82	<0.0001
Language	-0.0004	0.03	-0.02	0.98
Emotion x Language	-0.04	0.02	-2.01	0.05
Sentence Composability	-0.03	0.03	-1.12	0.27
Averaged LogFrequency	-0.02	0.08	-0.23	0.82

### Pupil size

**Fig 1:**
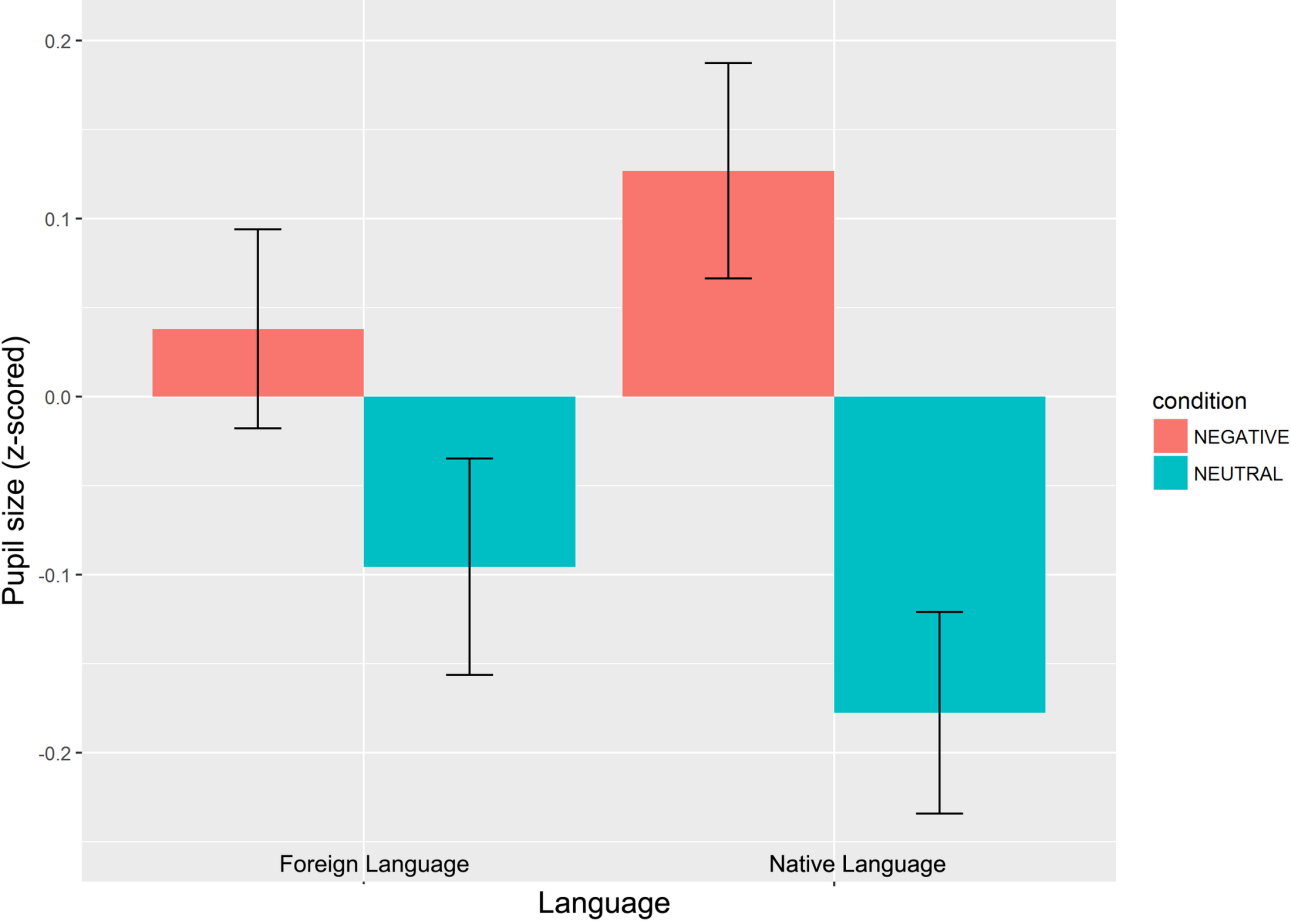
Z-scored pupil size means for Native and Foreign Language groups, in both Negative and Neutral Sentence conditions. Error bars refer to SEM calculated within participant.

The linear mixed-effect model to analyze pupillary responses was structured as for the subjective ratings, with the only exception that we had to drop the random slope for Emotion by participant because the amount of data was not sufficient for the model to converge. In order to standardize pupil sizes, each data point was z-scored per participant across the two Emotion conditions. Results showed an overall effect of Emotion (*t* = 4.82, *p* < .0001, *β* = 0.11 (0.02)) with pupil sizes being significantly greater when reading negative sentences (mean = 1409, SD = 345) than neutral ones (mean = 1394, SD = 344). Importantly, the main effect of Language was not significant (*t* = -0.02, *p*>.95, *β* = -0.0004 (0.02)), but there was a significant interaction between Emotion and Langauge (*t* = -2.01, *p* = .05, *β* = -0.04 (0.02)), suggesting differences in the magnitude of the effects between the two language contexts (see Table 4, Table 5B and Fig 1). None of the covariates led to significant effects (*ts*<-1.12, *ps*>.42). Follow-up least-squares means analyses confirmed that pupil sizes were greater for negative than for neutral sentences in both language groups, but that the Emotion effect was larger in the native than in the foreign language (native: [*t* = 4.91, *p* < .0001, *β* = 0.31 (0.06)]; foreign: [*t* = 2.22, *p* = .03, *β* = 0.14 (0.06)]).

## Discussion

We explored the effect of language (native vs. foreign) when reading neutral and emotional sentences on participants’ behavioral and implicit psychophysiological responses. Participants read sentences withnegative words and sentences including low arousing and neutrally valenced words. We observed an effect of emotion in participants’pupillary responses and explicit ratings. Emotional sentences resulted in augmented pupil dilations and higher emotional ratings as compared to neutral sentences. Furthermore, the effect of emotion on pupil dilations was smaller (namely, reduced by half) when participants read the sentences in the foreign language as compared to when they did so in the native one. Importantly, the main effect of language was far from significant This reveals that the different magnitude of the emotional effect in the two languages does not originate from cognitive dissimilarities driven by reading sentences in the native versus the foreign language. Together, these results provide compelling evidence in support of a certain degree of automatic emotional distance fostered by the foreign language, as previously suggested by introspective and experimental data yielding similar conclusions (see [29] for a review). The present study, however, went beyond previous ones in several ways. Firstly, it assessed online emotional reactivity to complex linguistic materials which did not tap into autobiographical memories. Additionally, the stimuli consisted of several emotional vs. neutral sentential contexts, contrary to the majority of the studies with single emotional words or noun phrases in isolation (e.g., [8]; [10]). Secondly, this research focused on disentangling implicit and explicit mechanisms of affective perception in bilinguals by using two different types of measures, with the purpose of sheding light on the potential source(s) of foreign language effects in emotion processing.

The difference found between the two linguistic contexts (foreign vs. native) was exclusively circumscribed to the automatic, emotion-mediated responses of the sympathetic nervous system, and it did not extend to the explicit behavioral ratings. This suggests that 1) highly automatic reactions to emotional verbal materials are lessened when the stimulus processing involves a foreign language, 2) that this emotional distance is mostly driven by a reduction in self-regulating affective mechanisms, and 3) that its origin may be independent of the difference in linguistic skills between native and foreign languages. The analysis of the ratings which explicitly evaluated the emotional impact of the materials, in fact, suggests that bilinguals from the two language groups were comparably aware of the difference between neutral and negative sentences, regardless of the language used. In other words, whatever the reasons of the reduced emotional resonance in foreign languages may be, we can confidently dismiss the idea that it is due to an impairment in the explicit perception of emotional content, and instead supports an automatic, and unconscious, weaker reactivity view (see also[14]).

In line with previous claims (see [6], for a review), we hypothesize that the locus of the emotional distance elicited by speaking in a foreign language relies on the precise contexts in which these languages are typically acquired. The emotionally neutral academic contexts in which foreign languages are generally learned, together with the lack of presence of such languages in the environment around the learners (namely, their defining property of being foreign), may be the cause of the weaker emotional reactivity reported in this and earlier studies. Contextual learning has been proposed as the mechanism through which emotional and linguistic information are bounded [4], and it is likely that learning a foreign language in a formal academic environment, as in the case of English for Spanish speakers living in Spain, does not provide provide opportunities for the creation of such strong bounds between linguistic and emotional content (see [11], for review).

To conclude, the present study shows that, in spite of the cost associated with dealing with a nonnative foreign language, the explicit understanding of emotional content is not impaired or distorted as compared to native languages. Emotional ratings provided by participants confirmed that they were consistently and fully aware of the emotional content carried by the sentences, regardless of the language. Yet, autonomic responses elicited by reading aloud emotionally-charged materials were reduced in the foreign language group providing evidence for an automatic emotional distance. We suggest that it is due to the fact that learning a language in an emotionally-grounded context favours the link between language and emotional processing, which boosts the affective resonance in native but not in foreign languages. This is certainly a tentative hypothesis deserving further research.

## Supporting information

S1 Fig Empathy questionnaire, Spanish version. (DOCX)
