# Supplementary material for: What do your eyes reveal about your foreign language? Reading emotional sentences in a native and foreign language
Source: PLoS One. 2017 Oct 3;12(10):e0186027. doi: 10.1371/journal.pone.0186027 (PMC5626519; doi:10.1371/journal.pone.0186027)
Supplement: S1 Fig — (DOCX) [file pone.0186027.s001.docx]

# ESCALA DE CONDUCTA CAMBRIDGE

Por favor, complete la siguiente información y luego lea las instrucciones de abajo.

**ESTA INFORMACION ES ESTRICTAMENTE CONFIDENCIAL**

Nombre:............................................Sexo:..............................................

Fecha de nacimiento:...................... Fecha de hoy :................................

**Cómo rellenar este cuestionario:**

Abajo hay una lista con frases. Por favor lea cuidadosamente cada una y juzgue en qué medida está usted de acuerdo o desacuerdo, marcando con un círculo su respuesta. No hay respuestas correctas ni incorrectas ni engañosas.

**PARA QUE ESTA ESCALA TENGA VALIDEZ TODAS LAS RESPUESTAS DEBEN ESTAR CONTESTADAS.**

*Ejemplos*

| Ej.1 Me enfadaría si no pudiera escuchar música todos los días. | Totalmente de acuerdo | Bastante de acuerdo | Un poco de acuerdo | Totalmente desacuerdo |
| --- | --- | --- | --- | --- |
| Ej.2 Prefiero hablar con mis amigos por teléfono que escribirles cartas. | Totalmente de acuerdo | Bastante de acuerdo | Un poco de acuerdo | Totalmente desacuerdo |
| Ej. 3 No me interesa viajar a diferentes partes del mundo. | Totalmente de acuerdo | Bastante de acuerdo | Un poco de acuerdo | Totalmente desacuerdo |
| Ej. 4 Prefiero leer que bailar. | Totalmente de acuerdo | Bastante de acuerdo | Un poco de acuerdo | Totalmente desacuerdo |
| 1. Me puedo dar cuenta fácilmente si alguien quiere entrar en una conversación. | Totalmente de acuerdo | Bastante de acuerdo | Un poco de acuerdo | Totalmente desacuerdo |
| 2. Prefiero los animales a las personas. | Totalmente de acuerdo | Bastante de acuerdo | Un poco de acuerdo | Totalmente desacuerdo |
| 3. Intento seguir las tendencias y modas actuales | Totalmente de acuerdo | Bastante de acuerdo | Un poco de acuerdo | Totalmente desacuerdo |
| 4. Cuando otros no me entienden a la primera me cuesta explicarles las cosas que para mi son fáciles de entender. | Totalmente de acuerdo | Bastante de acuerdo | Un poco de acuerdo | Totalmente desacuerdo |
| 5. Sueño la mayoría de las noches. | Totalmente de acuerdo | Bastante de acuerdo | Un poco de acuerdo | Totalmente desacuerdo |
| 6. Realmente me agrada cuidar de otras personas. | Totalmente de acuerdo | Bastante de acuerdo | Un poco de acuerdo | Totalmente desacuerdo |
| 7. Intento resolver mis propios problemas en lugar de discutirlos con otras personas. | Totalmente de acuerdo | Bastante de acuerdo | Un poco de acuerdo | Totalmente desacuerdo |
| 8. Me resulta difícil saber qué debo hacer en situaciones sociales. | Totalmente de acuerdo | Bastante de acuerdo | Un poco de acuerdo | Totalmente desacuerdo |
| 9. Cuando mejor estoy es al principio de la mañana. | Totalmente de acuerdo | Bastante de acuerdo | Un poco de acuerdo | Totalmente desacuerdo |
| 10. La gente a menudo me dice que defiendo con demasiada vehemencia mi punto de vista en una discusión. | Totalmente de acuerdo | Bastante de acuerdo | Un poco de acuerdo | Totalmente desacuerdo |
| 11. No me preocupa demasiado llegar tarde a una cita con un amigo. | Totalmente de acuerdo | Bastante de acuerdo | Un poco de acuerdo | Totalmente desacuerdo |
| 12. La amistad y las relaciones sociales son tan difíciles para mí que tiendo a no darles importancia. | Totalmente de acuerdo | Bastante de acuerdo | Un poco de acuerdo | Totalmente desacuerdo |
| 13. Nunca violaría la ley, aunque se tratase de algo sin importancia. | Totalmente de acuerdo | Bastante de acuerdo | Un poco de acuerdo | Totalmente desacuerdo |
| 14. A menudo me resulta difícil juzgar si alguien es maleducado o educado. | Totalmente de acuerdo | Bastante de acuerdo | Un poco de acuerdo | Totalmente desacuerdo |
| 15. En una conversación suelo centrarme en mis pensamientos en lugar de lo que puede estar pensando el otro. | Totalmente de acuerdo | Bastante de acuerdo | Un poco de acuerdo | Totalmente desacuerdo |
| 16. Prefiero los chistes sencillos más que los de ironía y sarcasmo. | Totalmente de acuerdo | Bastante de acuerdo | Un poco de acuerdo | Totalmente desacuerdo |
| 17. Vivo la vida pensando en el presente en lugar de en el futuro. | Totalmente de acuerdo | Bastante de acuerdo | Un poco de acuerdo | Totalmente desacuerdo |
| 18. Cuando era niño me gustaba cortar gusanos para ver qué ocurría. | Totalmente de acuerdo | Bastante de acuerdo | Un poco de acuerdo | Totalmente desacuerdo |
| 19. Capto rápidamente cuando alguien dice algo pero quiere decir otra cosa | Totalmente de acuerdo | Bastante de acuerdo | Un poco de acuerdo | Totalmente desacuerdo |
| 20. Suelo tener fuertes opiniones acerca de cuestiones relacionadas con la moral. | Totalmente de acuerdo | Bastante de acuerdo | Un poco de acuerdo | Totalmente desacuerdo |
| 21. Me cuesta entender por qué algunas cosas enfadan tanto a las personas. | Totalmente de acuerdo | Bastante de acuerdo | Un poco de acuerdo | Totalmente desacuerdo |
| 22. Me resulta fácil ponerme en el lugar de otra persona. | Totalmente de acuerdo | Bastante de acuerdo | Un poco de acuerdo | Totalmente desacuerdo |
| 23. Creo que los buenos modales es la cosa más importante que los padres pueden enseñar a sus hijos. | Totalmente de acuerdo | Bastante de acuerdo | Un poco de acuerdo | Totalmente desacuerdo |
| 24. Me gusta hacer las cosas espontáneamente. | Totalmente de acuerdo | Bastante de acuerdo | Un poco de acuerdo | Totalmente desacuerdo |
| 25. Soy bueno prediciendo como se sentirá alguien. | Totalmente de acuerdo | Bastante de acuerdo | Un poco de acuerdo | Totalmente desacuerdo |
| 26. Puedo reconocer en seguida cuando, en un grupo de gente, alguien se siente raro o incómodo. | Totalmente de acuerdo | Bastante de acuerdo | Un poco de acuerdo | Totalmente desacuerdo |
| 27. Si digo algo y alguien se siente ofendido pienso que es su problema y no el mío. | Totalmente de acuerdo | Bastante de acuerdo | Un poco de acuerdo | Totalmente desacuerdo |
| 28. Si alguien me pregunta si me gusta su corte de pelo yo respondo con la verdad incluso si no me gusta. | Totalmente de acuerdo | Bastante de acuerdo | Un poco de acuerdo | Totalmente desacuerdo |
| 29. No siempre puedo entender por qué alguien se puede sentir ofendido por un comentario. | Totalmente de acuerdo | Bastante de acuerdo | Un poco de acuerdo | Totalmente desacuerdo |
| 30. La gente a menudo me dice que soy impredecible. | Totalmente de acuerdo | Bastante de acuerdo | Un poco de acuerdo | Totalmente desacuerdo |
| 31. Disfruto siendo el centro de atención en una reunión social. | Totalmente de acuerdo | Bastante de acuerdo | Un poco de acuerdo | Totalmente desacuerdo |
| 32. Ver a la gente llorar no me pone triste. | Totalmente de acuerdo | Bastante de acuerdo | Un poco de acuerdo | Totalmente desacuerdo |
| 33. Me gusta discutir acerca de política. | Totalmente de acuerdo | Bastante de acuerdo | Un poco de acuerdo | Totalmente desacuerdo |
| 34. Soy muy directo, lo que mucha gente considera grosero incluso si lo hago sin esa intención | Totalmente de acuerdo | Bastante de acuerdo | Un poco de acuerdo | Totalmente desacuerdo |
| 35. No suelo encontrar las situaciones sociales confusas. | Totalmente de acuerdo | Bastante de acuerdo | Un poco de acuerdo | Totalmente desacuerdo |
| 36. La gente me dice que soy bueno comprendiendo como se siente y qué están pensando. | Totalmente de acuerdo | Bastante de acuerdo | Un poco de acuerdo | Totalmente desacuerdo |
| 37. Cuando hablo con la gente tiendo a hablar de sus experiencias más que de las mías. | Totalmente de acuerdo | Bastante de acuerdo | Un poco de acuerdo | Totalmente desacuerdo |
| 38. Me pone triste ver un animal sufriendo. | Totalmente de acuerdo | Bastante de acuerdo | Un poco de acuerdo | Totalmente desacuerdo |
| 39. Soy capaz de tomar decisiones sin la influencia de los sentimientos de los demás. | Totalmente de acuerdo | Bastante de acuerdo | Un poco de acuerdo | Totalmente desacuerdo |
| 40. No me puedo relajar hasta hacer la última cosa que tenía planeada para ese día. | Totalmente de acuerdo | Bastante de acuerdo | Un poco de acuerdo | Totalmente desacuerdo |
| 41. Puedo fácilmente decir si alguien está interesado o aburrido con lo que estoy diciendo. | Totalmente de acuerdo | Bastante de acuerdo | Un poco de acuerdo | Totalmente desacuerdo |
| 42. Me pongo triste si veo en las noticias gente sufriendo. | Totalmente de acuerdo | Bastante de acuerdo | Un poco de acuerdo | Totalmente desacuerdo |
| 43. Mis amigos suelen contarme sus problemas porque dicen que soy muy comprensivo. | Totalmente de acuerdo | Bastante de acuerdo | Un poco de acuerdo | Totalmente desacuerdo |
| 44. Puedo sentir cuando estoy siendo poco discreto sin necesidad de que me lo digan. | Totalmente de acuerdo | Bastante de acuerdo | Un poco de acuerdo | Totalmente desacuerdo |
| 45. Suelo empezar nuevos hobbies pero me aburro rápidamente de ellos y empiezo otra cosa. | Totalmente de acuerdo | Bastante de acuerdo | Un poco de acuerdo | Totalmente desacuerdo |
| 46. La gente a veces me dice que he ido demasiado lejos con las bromas. | Totalmente de acuerdo | Bastante de acuerdo | Un poco de acuerdo | Totalmente desacuerdo |
| 47. Me pondría muy nervioso subirme a una montaña rusa grande. | Totalmente de acuerdo | Bastante de acuerdo | Un poco de acuerdo | Totalmente desacuerdo |
| 48. La gente me dice a menudo que soy insensible, aunque no entiendo siempre por qué. | Totalmente de acuerdo | Bastante de acuerdo | Un poco de acuerdo | Totalmente desacuerdo |
| 49. Cuando hay alguien nuevo en el grupo considero que son los demás los que tienen que esforzarse para incluirle. | Totalmente de acuerdo | Bastante de acuerdo | Un poco de acuerdo | Totalmente desacuerdo |
| 50. Normalmente no me mantengo emocionalmente estable cuando veo una película. | Totalmente de acuerdo | Bastante de acuerdo | Un poco de acuerdo | Totalmente desacuerdo |
| 51. Me gusta ser organizado en la vida diaria y a menudo hago listas con las actividades que tengo que hacer. | Totalmente de acuerdo | Bastante de acuerdo | Un poco de acuerdo | Totalmente desacuerdo |
| 52. Sintonizo rápida e intuitivamente con cómo se siente otra persona. | Totalmente de acuerdo | Bastante de acuerdo | Un poco de acuerdo | Totalmente desacuerdo |
| 53. No me gusta asumir riesgos. | Totalmente de acuerdo | Bastante de acuerdo | Un poco de acuerdo | Totalmente desacuerdo |
| 54. En seguida me doy cuenta de que quiere hablar la otra persona. | Totalmente de acuerdo | Bastante de acuerdo | Un poco de acuerdo | Totalmente desacuerdo |
| 55. Puedo darme cuenta si alguien enmascara sus verdaderos sentimientos. | Totalmente de acuerdo | Bastante de acuerdo | Un poco de acuerdo | Totalmente desacuerdo |
| 56. Antes de tomar una decisión siempre considero los pros y los contras. | Totalmente de acuerdo | Bastante de acuerdo | Un poco de acuerdo | Totalmente desacuerdo |
| 57. No necesito pensar conscientemente las normas sociales de cada situación. | Totalmente de acuerdo | Bastante de acuerdo | Un poco de acuerdo | Totalmente desacuerdo |
| 58. Soy bueno prediciendo que hará la gente | Totalmente de acuerdo | Bastante de acuerdo | Un poco de acuerdo | Totalmente desacuerdo |
| 59. Tiendo a involucrarme emocionalmente en los problemas de mis amigos. | Totalmente de acuerdo | Bastante de acuerdo | Un poco de acuerdo | Totalmente desacuerdo |
| 60. Normalmente respeto el punto de vista del otro, aunque no lo comparta. | Totalmente de acuerdo | Bastante de acuerdo | Un poco de acuerdo | Totalmente desacuerdo |

***Gracias por completar este cuestionario.***

© MRC-SBC/SJW Feb 1999
